# ORMDL3 regulates poly I:C induced inflammatory responses in airway epithelial cells

**DOI:** 10.1186/s12890-021-01496-5

**Published:** 2021-05-17

**Authors:** Gemma Laura, Yi Liu, Kieran Fernandes, Saffron A. G. Willis-Owen, Kazuhiro Ito, William O. Cookson, Miriam F. Moffatt, Youming Zhang

**Affiliations:** 1 National Heart and Lung Institute, Imperial College London, London, SW3 6LY UK; 2 Pulmocide Ltd., London, WC2A 1AP UK

**Keywords:** ORMDL3, Poly I:C, TLR3, Epithelial cell, Inflammatory response

## Abstract

**Background:**

Oroscomucoid 3 (ORMDL3) has been linked to susceptibility of childhood asthma and respiratory viral infection. Polyinosinic-polycytidylic acid (poly I:C) is a synthetic analog of viral double-stranded RNA, a toll-like receptor 3 (TLR3) ligand and mimic of viral infection.

**Methods:**

To investigate the functional role of ORMDL3 in the poly I:C-induced inflammatory response in airway epithelial cells, *ORMDL3* knockdown and over-expression models were established in human A549 epithelial cells and primary normal human bronchial epithelial (NHBE) cells. The cells were stimulated with poly I:C or the Th17 cytokine IL-17A. IL-6 and IL-8 levels in supernatants,  mRNA levels of genes in the TLR3 pathway and inflammatory response from cell pellets were measured. *ORMDL3* knockdown models in A549 and BEAS-2B epithelial cells were then infected with live human rhinovirus (HRV16) followed by IL-6 and IL-8 measurement.

**Results:**

*ORMDL3* knockdown and over-expression had little influence on the transcript levels of *TLR3* in airway epithelial cells. Time course studies showed that *ORMDL3*-deficient A549 and NHBE cells had an attenuated IL-6 and IL-8 response to poly I:C stimulation. A549 and NHBE cells over-expressing *ORMDL3* released relatively more IL-6 and IL-8 following poly I:C stimulation. IL-17A exhibited a similar inflammatory response in *ORMDL3* knockdown and over-expressing cells, but co-stimulation of poly I:C and IL-17A did not significantly enhance the IL-6 and IL-8 response. Transcript abundance of *IFNB* following poly I:C stimulation was not significantly altered by *ORMDL3* knockdown or over-expression. Dampening of the IL-6 response by *ORMDL3* knockdown was confirmed in HRV16 infected BEAS-2B and A549 cells.

**Conclusions:**

ORMDL3 regulates the viral inflammatory response in airway epithelial cells via mechanisms independent of the TLR3 pathway.

**Supplementary Information:**

The online version contains supplementary material available at 10.1186/s12890-021-01496-5.

## Background

Asthma is a syndrome defined by symptoms of reversible airway inflammation, intermittent wheeze and shortness of breath, and caused by a combination of genetic, epigenetic and environmental factors. Acute exacerbations are the major cause of morbidity and mortality from the disease and severe asthma remains difficult to treat due to lack of effective therapeutic options. In 2007, a genome-wide association study (GWAS) of asthma identified single nucleotide polymorphisms (SNPs) flanking *ORMDL3* gene on chromosome 17 to be significantly associated with the disease [1]. This association then has subsequently been replicated in many studies worldwide, including a multi-ancestry global meta-analysis [2]. Chromosome 17 *ORMDL3* locus is now recognised as the major predisposing factor for childhood-onset asthma. Significant increases in the number of wheezing illnesses have been observed in children with enhanced transcription genotypes at the 17q21 *ORMDL3 *locus.

Rhinoviruses are the most frequent pathogen associated with the symptoms of virial respiratory infections (VRI) [3] and early symptomatic human rhinovirus (HRV) infection is a major risk factor for subsequent asthma. HRV infection accounts for nearly two thirds of childhood asthma exacerbations [4]. Evidence now indicates that the association between the 17q21 *ORMDL3* locus and childhood-onset asthma is also limited to those children with HRV-induced wheezing in early life [5], however the mechanism for this interaction remains unclear. We previously reported that *ORMDL3* has multiple effects on host–pathogen interactions, stress responses and ubiquitination in cells. Importantly, ORMDL3  regulates the major HRV receptor, intercellular adhesion molecule 1 (ICAM1) during the IL-1β-stimulated inflammatory response [6]. ORMDL3 was also found to promotes HRV replication in human epithelial cells [7]. Human ORMDL3 is a trans-membrane protein anchoring in the endoplasmic reticulum (ER). The ER is the site responsible for protein folding, synthesis of lipids and the storage of free calcium. ER stress can reduce the capacity of the ER for protein folding and thereby dictate cellular responses to inflammation. It interacts with the serine SPT enzyme complex in sphingolipid synthesis [8]. ORMDL3 facilitates the unfolded protein response to cellular stress by influencing sarcoplasmic/endoplasmic reticulum calcium ATPase (SERCA) and ER-mediated Ca^2+^ flux [9]. ORMDL3 also regulate endoplasmic reticulum (ER) stress, cermide and shingosine-1-sulfate levels that regulate cytokine release. ORMDL3 could work in multiple pathways in regulating HRV infections [10]. 

In this brief report, we investigated the role of ORMDL3 in the host inflammatory response to viral stimulation, as mimicked by polyinosinic-polycytidylic acid (poly I:C), in airway epithelial cells. Poly I:C is a synthetic analog of viral double-stranded RNA (dsRNA) and a mimic of viral infection. is recognized by toll-like receptor 3 (TLR3, also known as CD283) in a variety of cell types, including B-cells, macrophages, dendritic and epithelial cells  [11, 12], triggering an innate immune response and recapitulating the major cellular, physiological and molecular changes characteristic of the host inflammatory response to viral infection. These include a loss of epithelial integrity, increased production of mucus and induction of inflammatory cytokines, most notably, the major mediator of host antiviral defence; interferon (IFN)-ß [13]. Recent evidence indicates that IL-17A and poly I:C act synergistically to induce production of chemo-attractants CXCL1 and IL-8 [14] and release of inflammatory cytokines [15] in bronchial epithelial cells. We established *ORMDL3* knockdown and over-expression models in A549 epithelial and NHBE cells and stimulated the cells with either poly I:C or IL-17A alone or in combination. The inflammatory response measured over a 24-h time course for mono-stimulation experiments, and at 10 h for co-stimulation experiments through mRNA levels of key genes and protein levels of IL-6 and IL-8 in the supernatant. We finally tested the inflammatory cytokine release in *ORMDL3* knockdown A549 and BEAS-2B epithelial cells infected by live human rhinovirus (HRV16).

## Methods

### Cell culture and stimulations

Human lung epithelial cell line A549 cells were purchased from American Type Culture Collection (ATCC). The cells were cultured in Dulbecco’s Modified Eagle’s Medium (DMEM) containing 10% vol/vol (v/v) FBS and 2 mM L-Glutamine. A549 cells were maintained in 150 cm^2^ (T150) flasks at 37 °C with 5% CO_2_. The SV-40 immortalised human bronchial epithelial cell line BEAS-2B was purchased from American Type Culture Collection (ATCC, Manassas, VA, USA), maintained in LHC-8 medium (Invitrogen, Paisley, UK) at 37 °C, 5% CO_2_, and sub-cultured twice a week. Normal human bronchial epithelial (NHBE) cells were obtained from Lonza and cultured in human airway epithelial cell (hAEC) culture medium (Epithelix, Switzerland). Poly I:C was purchased from Sigma-Aldrich (cat P1530, UK). Recombinant human IL-17A was obtained from R&D (cat 317-ILB-050, UK). Following gene knockdown or over-expression and incubation with the respective culture medium in 24 well plates, A549 and NHBE cells were stimulated in triplicate with 10 μg/ml poly I:C per well or/with 100 μg/ml IL-17A per well.

### *ORMDL3* knockdown and overexpression

*ORMDL3* gene knockdown was performed by using gene specific *ORMDL3* siRNA ON-TARGETplus SMARTpool (Dharmacon Research Inc., Lafayette, CO). ON-TARGETplus Non-targeting Pool (Dharmacon Research Inc., Lafayette, CO) was used for control in the experiments. Transfection of 40,000 A549 cells each well was carried out with using Thermo Scientific DharmaFECT transfection reagent 4. In total 25 nM of siRNA in serum free medium (Sigma-Aldrich) was added to each well simultaneously and the cells were placed in a CO_2_ incubator at 37 °C for 48 h. The medium of each cell was removed, cells were starved in serum free medium for 24 h and then stimulated with poly I:C or IL-17A. For construction of an *ORMDL3* over-expression plasmid, we generated an *ORMDL3* gene specific PCR from control cDNA (Clonetech, USA). The sequences for primers were: forward, CACCATGAATGTGGGCACAGCGCACAGCGAG; reverse, TCAGTACTTATTGATTCCAAAAATC. The PCR product was then cloned to the pcDNATM3.1 directional TOPO^®^ expression vector (Invitrogen) according to the manufacturer's protocol. Clones containing the correct insert were sequenced and verified. For the over-expression experiment 250 ng of the *ORMDL3* over-expressing  plasmid or control plasmid with 1μl lipofectamine™ 2000 (Invitrogen) were used per well. Cells were cultured for 48 h, then the medium was removed and serum free medium was added for 24 h. Cells and supernatants were collected at 0, 6, 10, 16 and 24 h for time course studies. For co-stimulation of poly I:C and IL-17A, samples were collected at 10 h.

### Western blotting

For ORMDL3 expression, whole-cell protein extracts were prepared by using the RIPA Lysis Buffer (Millipore). For each sample, 50 μg of protein was separated by electrophoresis on 10% sodium dodecyl sulfate polyacrylamide gels (Invitrogen) and transferred to nitrocellulose membranes using the iBlot™ DryBlotting device (Invitrogen) and iBlot™ Transfer stacks. After blocking for 1 h with 5% milk, the blot was immersed with the first antibody (1: 500, Rabbit anti-human ORMDL3, ABGENT Cat no: AP10739c) and then detected using secondary antibodies conjugated to horseradish peroxidase (HRP) (1:4000; DAKO Cytomation) and enhanced chemi-luminescence GE Healthcare ECL/ECL Plus (Amersham) detection solutions.

### Enzyme-linked immunosorbent assays (ELISA)

Cell-free supernatants were harvested and stored for further cytokine measurements. Human IL-6 and IL-8 were measured using ELISA kits according to the manufacturer’s instructions (PeproTech, US).

### Quantitative PCRs

RNA was extracted from samples using the protocol and RNeasy Plus Mini Kit provided by Qiagen (Qiagen, UK). Extracted RNA was transcribed into complementary DNA (cDNA) using a High-Capacity cDNA Reverse Transcription Kit (Applied Biosystems, Lithuania). qPCRs were performed in QuantStudio 7 Flex Real-Time PCR System with KAPA SYBR® FAST Universal (Sigma-Aldrich). Forward and reverse primer sequences are: *GAPDH, CACCATCTTCCAGGAGCGAG, CCTTCTCCATGGTGGTGAAGAC; ORDML3,TTGTGAGTGTCCCTGTCG, AGTGTAGAAGCTGGTGAGG; TLR3, AGAGTTGTCATCGAATCAAATTAAAG, AATCTTCCAATTGCGTGAAAA; TRIF, CCGGATCCCTGATCTGCTTG, ATGTCGAAGGCGCTAGGAAG; IFNB, GATTCATCTAGCACTGGCTGG, CTTCAGGTAATGCAGAATCC; IL1,TCTTTCTGGCTTAGAACAAAGGGGC, AGTAAAGGTAGCCCTTGTTTCCCCC; IL6, TACCCCCAGGAGAAGATTCC, GCCATCTTTGGAAGGTTCAG; IL8, ACTGAGAGTGATTGAGAGTGGAC, AACCCTCTGCACCCAGTTTT.*

### *TLR3* transcript levels in microarray data from *ORMDL3* knockdown A549 cells

We examined *TLR3* transcript levels in our previously published global gene profiling of *ORMDL3* knockdown in A549 cells. The methods, protocols and analysis methods are described in [6].

### HRV16 infection *in ORMDL3* knockdown A549 and BEAS-2B cells

Human rhinovirus (HRV16) (ATCC ®VR-283™) was purchased from American tissue culture collection (Manassas, VA, USA), and propagated in Hela cells (ATCC®CRL-1958™). A549 cells or BEAS-2B cells were transfected with *ORMDL3 *siRNA or scramble oligonucleotide as shown above and incubated for 4 days at 37 °C, 5% CO_2_. HRV16 (5 multiplicity of infection, MOI) was then applied to cells and incubated for 48 h at 33 °C, 5% CO_2_. The cell-free supernatant was harvested and stored for IL-6 and IL-8 measurements. Human IL-6 and IL-8 was measured using ELISA kits as shown above. IL-6 and IL-8 levels of non-infection cells were applied to be compared in each group.

### Statistical analysis

Human IL-6 and IL-8 arithmetic means were compared by one-way analysis of variance [1]. All statistical analyses were performed, and graphs drawn using GraphPad Prism Version 5.01 software (GraphPad Software Inc, California, USA). Quantitative real-time PCR (qPCR) was conducted using QuantStudio 7 Flex Real-Time PCR System (Applied Biosystems). Measurements were ascertained in triplicate for each gene (*ORDML3*, *TLR3*, *TRIF*, *IFNB, IL1*, *IL6*, *IL8*), with *GAPDH* as the reference gene. The comparisons of ΔΔCT of each experiment groups were performed with Student’s *t* tests. Data presented are averages ± SEM (standard error of the mean). All statistical hypothesis tests were two tailed and a *P* value of less than 0.05 was considered significant.

## Results

### *ORMDL3* does not impact baseline *TLR3* expression in A549 cells

**Fig.1:**
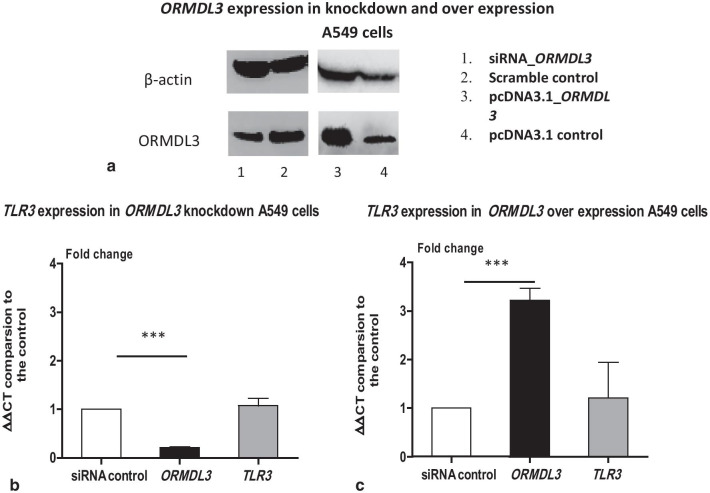
TLR levels in *ORMDL3* knockdown and over-expressing  A549 cells. A549 cells were seeded at 40,000 cell/per well in 24 well culture plate. **a** Western Blotting images of ORMDL3 expression in knockdown and over-expressing  A549 cells. Full-length blots were presented in Additional file 1:  Western Bollting Images of ORMDL3. **b** 25 nM siRNA control and *ORMDL3* siRNA were added into the cell culture medium. The cells were cultured for 48 h. **c** 250 ng/ml *pcDNA3.1-ORMDL3* plasmid and control plasmid were added into the cell culture medium. Transcript levels of *ORMDL3, TLR3* were quantified by qPCR and with *GAPDH* as internal control. ΔΔCT were compared with siRNA control samples. Student’s *t* test was calculated. ***Indicates *P* < 0.001. Each group contained triplicated experiments

*TLR3* transcript abundance data were available as part of a prior global gene expression time course study of *ORMDL3* knockdown and IL-1β stimulation in A549 cells [6]. *TLR3*, as indexed by Affymetrix Human Gene 1.1 ST transcript cluster 8,098,611, showed no significant difference in baseline gene expression dependent on *ORMDL3* knockdown status (control group expression 7.21, knockdown group 7.48, *P* = 5.60 × 10^–02^). Looking across the time-course, *TLR3* showed a small, nominally significant difference in response to IL-1β stimulation between control cells and cells deficient for *ORMDL3* (F = 3.27, *P* = 2.31 × 10^–02^), though significance was not retained following a global *P *value adjustment. The transcript abundance of *TLR3* in *ORMDL3* knockdown and control cells is listed in Table 1. The results indicate that *ORMDL3* may have a minor influence on *TLR3* expression, detectable only under stimulation. To confirm absence of a baseline difference we used siRNA to silence *ORMDL3* and the *pcDNA3.1-ORMDL3* plasmid to generate over-expression of *ORMDL3* in A549 cells (Fig. 1a). We examined *TLR3* transcript levels in the cells. The transcript abundance of *TLR3* did not differ significantly between cells over-expressing or deficient for *ORMDL3*. Together these data suggest that *ORMDL3* expression has no significant influence of the baseline expression of *TLR3* in epithelial cells (shown in Fig. 1b, c).

**Table 1:** The *TLR3* transcript levels in *ORMDL3* knockdown A549 cells during IL1b stimulation

Time points	Group	Avr. exp	Comparison	Fold change	Q value	Comparison	Fold change	Q value
0 h	Control	7.21	–			–		
2 h	Control	7.40	2 h/0 control	1.15	0.35	–		
4 h	Control	7.38	4 h/0 control	1.14	0.24	–		
8 h	Control	7.10	8 h/0 control	0.89	0.30	–		
10 h	Control	6.98	10 h/0 control	0.87	0.22	–		
0 h	siRNA	7.48	–			0 h siRNA/control	1.24	0.27
2 h	siRNA	7.15	2 h/0 siRNA	0.78	0.03	2 h siRNA/control	0.83	0.26
4 h	siRNA	7.62	4 h/0 siRNA	1.06	0.59	4 h siRNA/control	1.16	0.81
8 h	siRNA	7.19	8 h/0 siRNA	0.81	0.06	8 h siRNA/control	1.12	0.67
10 h	siRNA	7.17	10 h/0 siRNA	0.79	0.06	10 h siRNA/control	1.14	0.81

Total 25 nM *ORMDL3* siRNA and control were transfected into A549 cells. RNAs were extracted from the cells after IL-1B (1 ng/ml) stimulations at 0, 2, 4, 8 and 10 h. Global gene profiling was performed by using Affymetrix Human Gene 1.1 ST Arrays. 0 h, 2 h, 4 h, 8 h,10 h indicate the sample collecting time after IL-1B stimulation

*Avr. Exp* average expression; h: hour

### *ORMDL3* regulates the poly I:C-induced inflammatory response in A549 cells

**Fig.2:**
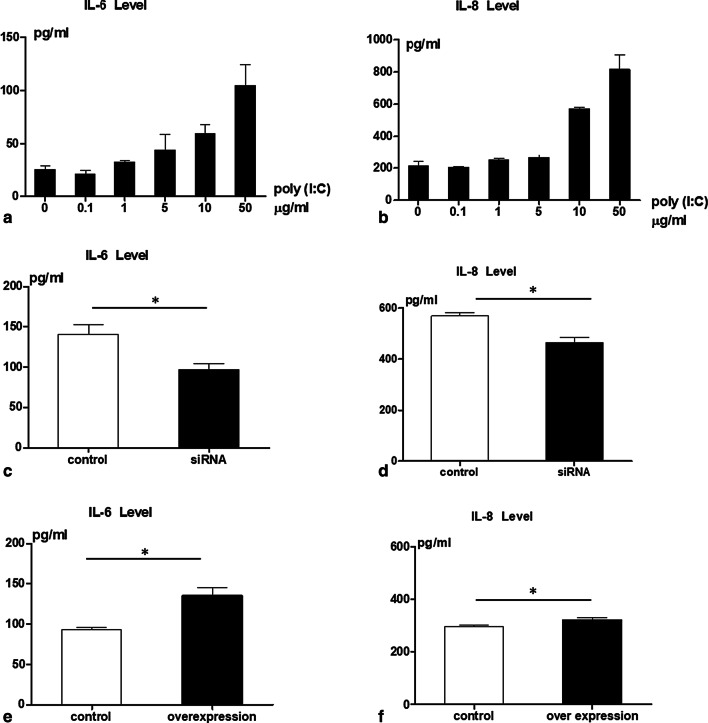
Inflammatory response induced by poly I:C in A549 cells. A549 cells were seeded with full medium for 24 h, then changed to serum-free medium for 1 day. Poly I:C was added to the wells at different concentrations. Supernatants were collected for measurements for IL-6 and IL8 levels after 10 h. **a** The IL-6 levels of poly I:C titration, **b** the IL-8 levels of poly I:C titration. **c** The IL-6 levels in *ORMDL3* knockdown cells after poly I:C stimulation for 10 h. **d** The IL-8 levels after *ORMDL3* knockdown cells after poly I:C stimulation for 10 h. **e** The IL-6 levels in *pcDNA3.1-ORMDL3* over-expressing cells after poly I:C stimulation for 10 h. **f** The IL-8 levels after *pcDNA3.1-ORMDL3* over-expressing cell after poly I:C stimulation for 10 h.

In order to determine whether poly I:C can induce an inflammatory response in human airway epithelial cells we stimulated A549 cells with poly I:C at concentrations of 0, 1, 1, 5, 10 and 50 μg /ml for 10 h. Poly I:C induced an inflammatory response in a dose dependent manner with supernatant IL-6 and IL-8 levels increasing with each poly I:C dosage increment (shown in Fig. 2a, b). We monitored the cell viability at each poly I:C dosage concentration. Poly I:C affected the cell viability at a concentration of 50 μg/ml (data not shown). We therefore applied a concentration of 10 μg/ml for all further experiments. In order to assess participation of *ORMDL3* in the poly I:C-induced inflammatory response we stimulated *ORMDL3*-deficient and *ORMDL3* over-expressing A549 cells with poly I:C for 10 h. *ORMDL3* knockdown resulted in a blunted inflammatory response to poly I:C, with relatively reduced IL-6 and IL-8  release at 10 h (*P* < 0.05 respectively; Fig. 2c, d). Conversely *ORMDL3* over-expression yielded an exaggerated inflammatory response, with poly I:C inducing relatively higher levels of IL-6 and IL-8 release (*P* < 0.05 respectively, Fig. 2e, f). These results confirm that poly I:C induces an inflammatory response in airway epithelial cells and identify *ORMDL3* as a factor regulating the magnitude of this response.

### Time course studies of poly I:C stimulation in A549 and NHBE cells

**Fig.3:**
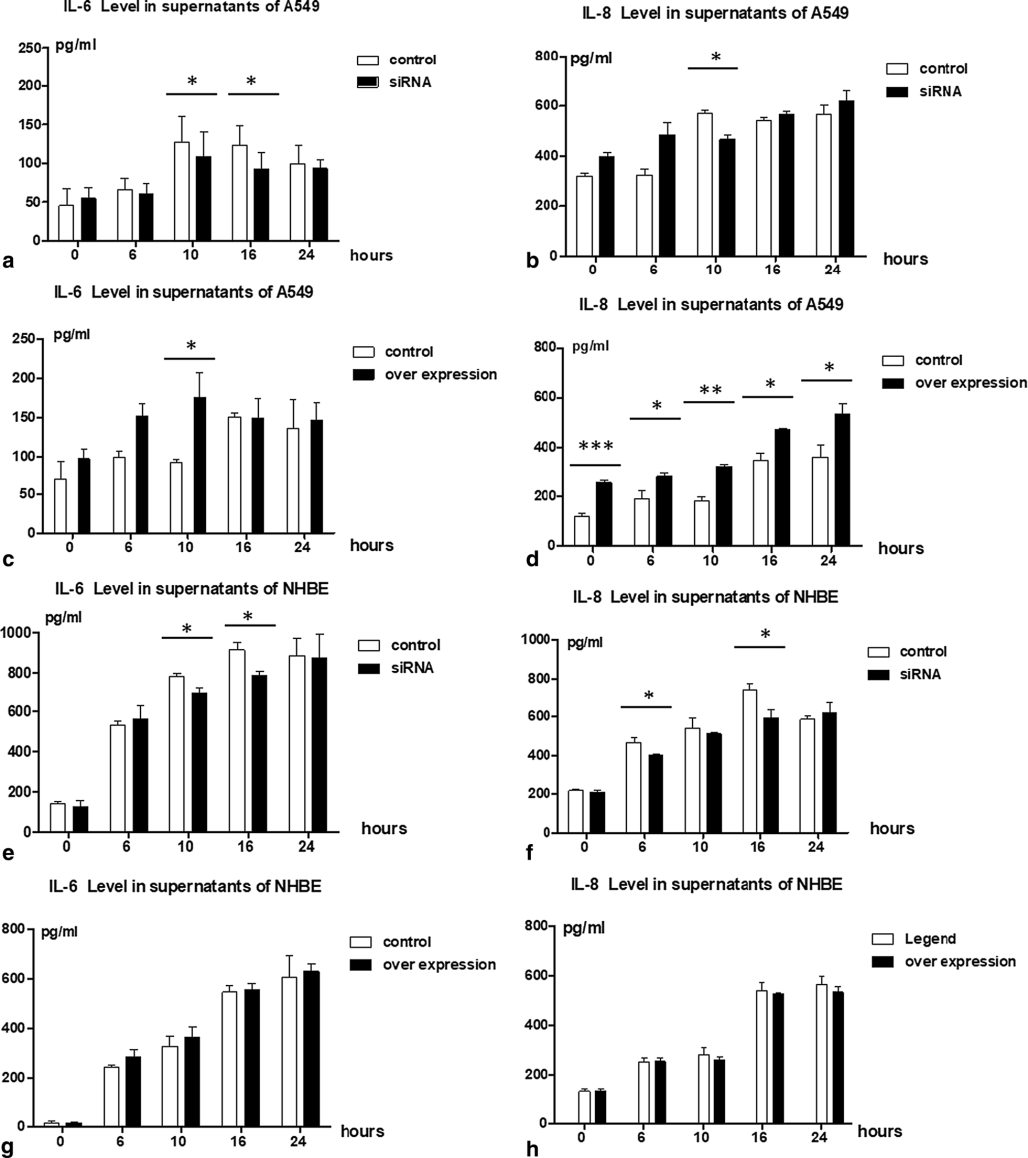
Time course studies in epithelial cells stimulated with poly I:C. A549 cells and NHBE cells were used for time course studies. Cells were transfected with 25 nM *ORMDL3* siRNA or 250 ng/ml *pcDNA3.1-ORMDL3* for 2 days, then changed to serum free medium. Cells were stimulated with 10 μg/ml poly I:C for 6, 10, 16 and 24 h. The supernatants were collected. **a** The IL-6 levels in *ORMDL3* knockdown A549 cell samples. **b** The IL-8 levels in *ORMDL3* knockdown A549 cell samples. **c** The IL-6 levels in *ORMDL3* over-expressing  A549 cell samples. **d** The IL-8 levels in *ORMDL3* over-expressing  A549 cell samples. **e** The IL-6 levels in *ORMDL3* knockdown NHBE cell samples. **f** The IL-8 levels in *ORMDL3* knockdown NHBE cell samples. **g** The IL-6 levels in *ORMDL3* over-expressing  NHBE cell samples. **h** The IL-8 levels in *ORMDL3* over-expressing  A549 cell samples. Samples were analyzed by one way ANOVA. ***Indicates *P* < 0.001; **indicates *P* < 0.01; *indicates *P* < 0.05, each group contained triplicated experiments

To further explore the role of *ORMDL3* in the poly I:C inflammatory response we set up 24-h time-course studies of poly I:C stimulation in A549 and NHBE cells deficient or over-expressing for *ORMDL3*. After a 48-h  transfection cells were stimulated with poly I:C (10 μg/ml) and supernatant samples collected at 0, 6, 10, 16 and 24 h. IL-6 and IL-8 production in each condition was measured and compared to their respective controls. In A549 cells under conditions of *ORMDL3* knockdown IL-6 levels were relatively reduced at all time-points except 0 h (baseline), with statistical significance achieved at 10 h only (*P* < 0.05). A consistent decrease in release from *ORMDL3* knockdown cells was observed from 10 to 24 h . IL-8 levels were also significantly decreased at 10 h (*P* < 0.05) but showed no consistent trend at other time points (shown in Fig. 3a, b). For A549 cells over-expressing *ORMDL3* IL-6 levels were relatively increased at 6 h  but this difference achieved significance at 10 h only (*P* < 0.05, shown in Fig. 3c, d). For NHBE cells, O*RMDL3*-deficient cells released significantly less IL-6 at 10 and 16 h  after poly I:C stimulation (*P* < 0.05). IL-8 levels were also significantly lower at 6 and 16 h time-points compared to control (*P* < 0.05) (shown in Fig. 3e, f). For *ORMDL3* over expressing NHBE cells, whilst both IL-6 and IL-8 levels were elevated relative to control at all time points, these differences did not achieve statistical significance (shown in Fig. 3g, h).

Our results confirm a bidirectional influence of *ORMDL3* on the poly I:C inflammatory response in epithelial cells. Silencing of *ORMDL3* blunts the inflammatory response whilst over-expression increases its magnitude. The impact of *ORMDL3* manipulation on the poly I:C response was not however, as large as its impact on the IL-1β response as reported in our previous experiments [6].

### Co-stimulation of poly I:C and IL-17A in *ORMDL3* knockdown and over-expressing A549 cells

The Th17 cytokine IL-17A can enhance the poly I:C inflammatory response in epithelial cells [15]. We therefore investigated the impact of *ORMDL3* knockdown and over-expression under conditions of poly I:C and IL-17A co-stimulation in A549 cells.

**Fig.4:**
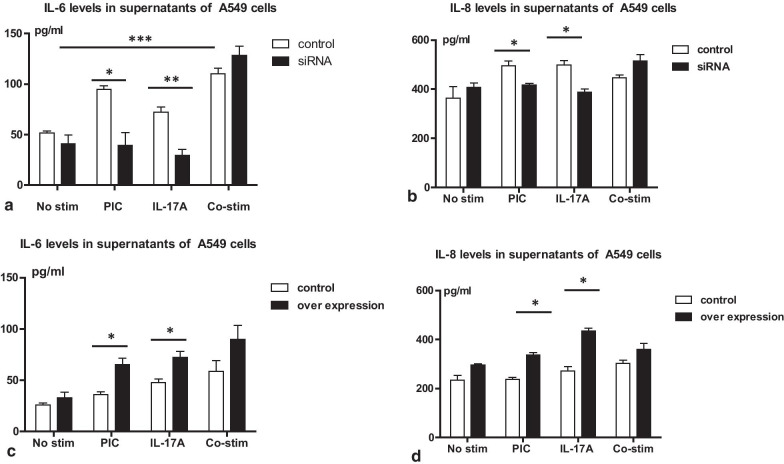
Co-stimulation with poly I:C and IL-17A in A549 cells. A549 cells were used for co-stimulations with 10 µg/ml poly I:C and 100 µg/ml IL-17A for 10 h in *ORMDL3* knockdown and *ORMDL*3 over-expression A549 cells for 10 h. **a** The IL-6 levels in *ORMDL3* knockdown A549 cell samples. **b** The IL-8 levels in *ORMDL3* knockdown A549 cells. **c** IL-6 levels in *ORMDL3* over-expressing  A549 cells. **d** IL-8 levels in *ORMDL3* over-expressing  A549 cell samples. Samples were analyzed by one way ANOVA. ***Indicates *P* < 0.001; ** indicates *P* < 0.01; *indicates *P* < 0.05, each group contained triplicated experiments. PIC: poly I:C, *Co-stim:* co-stimulation of poly I:C and IL-17A, *No-stim:* no-stimulation

In *ORMDL3*-deficient cells, as expected, poly I:C induced lower IL-6 levels at 10 h relative to control (*P* < 0.05). Likewise, IL-17A also induced lower IL-6 levels in *ORMDL3*-deficient cells relative to control (*P* < 0.01). Whilst co-stimulation of poly I:C with IL-17A enhanced the IL-6 response in control cells (*P* < 0.001), this response did not differ significantly between control and *ORMDL3* knockdown cells (*P* = 0.16, shown in Fig. 4a). Similarly, whilst both poly I:C and IL-17A yielded lower magnitude IL-8 responses in *ORMDL3* knockdown cells relative to control when applied independently (*P* < 0.05), no difference in IL-8 levels was seen between control and *ORMDL3* knockdown cells under poly I:C and IL-17A co-stimulation (*P* = 0.12, shown in Fig. 4b). In *ORMDL3* over expressing cells both poly I:C and IL-17A led to an elevation in IL-6 levels relative to control when applied independently (*P* < 0.05), however no difference was seen between control and *ORMDL3* over expressing cells under conditions of co-stimulation (*P* = 0.79, shown in Fig. 4c). Similarly, both poly I:C stimulation and IL-17A stimulation yielded a relatively larger IL-8 response in *ORMDL3* over-expressing cells relative to control when applied independently (*P* < 0.05), however no difference was observed between control and *ORMDL3* over expressing cells under conditions of co-stimulation (*P* = 0.46, shown in Fig. 4d).

These results show that *ORMDL3* influences the inflammatory response induced by IL-17A and poly I:C in epithelial cells. Manipulation of *ORMDL3* does not however, influence the response to co-stimulation, suggesting that *ORMDL3* may have different, non-additive roles in the signal transduction of TRL and Th17 pathways in epithelial cells.

### The transcript levels of *TLR3*, *IFNB*, *TRIF, IL1, IL6 and IL8 *in *ORMDL3* knockdown and over-expressing A549 cells after poly I:C stimulation

**Fig. 5:**
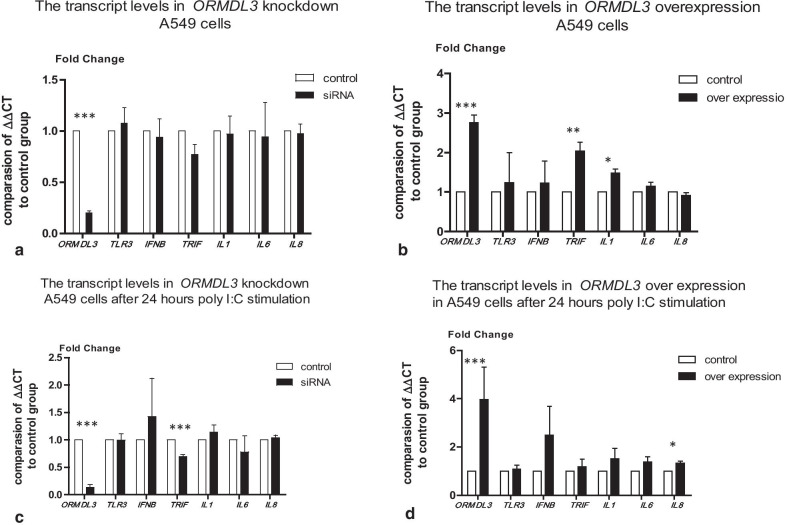
Gene transcript levels in *ORMDL3* knockdown and over-expression before and after poly I:C stimulation. *ORMDL3* knockdown or over-expression A549 cells and NHBE cells were stimulated with 10 μg/ml poly I:C for 24 h. RNAs were extracted and qPCRs were performed. **a** Gene transcript levels at *ORMDL3* knockdown cells  and control cells. **b** Gene transcript levels at *ORMDL3* over-expressing  cells and control cells. **c** Transcript levels at *ORMDL3* knockdown cells and control cells after 24 h stimulation with poly I:C. **d** Gene transcript levels in *ORMDL3* over-expressing cells  and control cells  after 24 h stimulations with poly I:C. ***indicates *P* < 0.001; **indicates *P* < 0.01; *indicates *P* < 0.05, each group contained triplicated experiments

To further delineate the role of *ORMDL3* we analysed the abundance of transcripts responsible for TLR3 signalling and the inflammatory response in A549 cells deficient for, or over-expressing *ORMDL3* at baseline, and following 10 µg/ml poly I:C stimulation for 24 h. IFNβ is type I class of interferon and is important for defence against viral infections. *TRIF* encodes an adaptor protein containing a toll/interleukin-1 receptor homology domain, which is an intracellular signalling domain that mediates protein–protein interactions between the TLRs and signal-transduction components. It specifically interacts with TLR3, but not with other TLRs, and this association mediates dsRNA induction of IFNβ through activation of nuclear factor kappa-B (NF-κB) during an antiviral immune response. We investigated the transcript levels of *TLR3, IFNB* and *TRIF* and the inflammatory cytokine genes *IL1, IL6* and *IL8*. In *ORMDL3* knockdown A549 cells at baseline, transcript levels of *TLR3*, *IFNB*, *TRIF, IL1, IL6 and IL8* showed no significant difference (shown in Fig. 5a). In *ORMDL3* over-expressing cells at baseline the transcript levels of *TRIF* and *IL1* were significantly raised relative to the control group (*P* < 0.01, *P* < 0.05 respectively, shown in Fig. 5b). We then compared transcript levels at 24 h  after stimulation. Surprisingly, we found that poly I:C significantly down regulated the TLR pathway genes *IFNB* and *TRIF*, and up regulated the cytokine genes *IL1*, *IL6* and *IL8* both after 24 h (*P* < 0.001 respectively, data not shown). Next, we compared the transcript levels in cells deficient for, or over-expressing *ORMDL3* at 24 h following stimulation. For *ORMDL3* knockdown cells, *TRIF* transcript levels were significantly lower than the control group (*P* < 0.001) (shown in Fig. 5c). For *ORMDL3* over expressing cells, although transcript levels for all genes were higher than the control group, only the *IL8* transcript achieved significance (*P* < 0.05) (shown in Fig. 5d). The results show that whilst *ORMDL3* did not significantly influence transcript levels of *TLR3* and *IFNB* after poly I:C stimulation, it did impact transcript levels of *TRIF* and several cytokine genes.

### The inflammatory response in *ORMDL3* knockdown epithelial cells after HRV-16 infection

**Fig.6:**
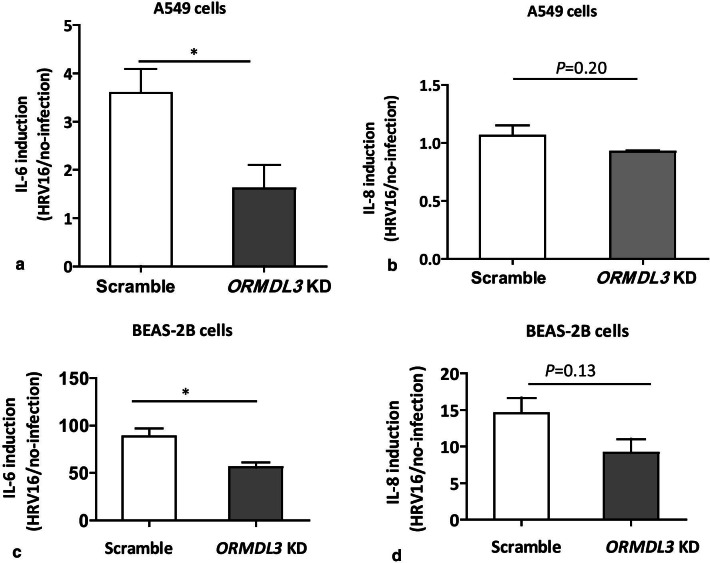
IL-6 and IL-8 levels in *ORDML3* knockdown A549 and BEAS-2B epithelial cells after HRV-16 infection. *ORMDL3* knockdown A549 and BEAS-2B cells were infected with HRV-16 (5 MOI) and incubated for 48 h at 33 °C, 5% CO_2_. The cell-free supernatant was harvested and both IL-6 and IL-8 were measured. IL-6 and IL-8 levels in non-infection cells were utilized as comparison in each group. **a** IL-6 induction levels in *ORMDL3* A549 cells after HRV-16 infection. **b** IL-8 induction levels in *ORMDL3* A549 cells after HRV-16 infection. **c** IL-6 induction levels in *ORMDL3* BEAS-2B cells after HRV-16 infection. **d** IL-8 induction levels in *ORMDL3* BEAS-2B cells after HRV-16 infection. *Indicates *P* < 0.05, each group contained triplicated experiments excepting experiment A had six experiments

In order to determine whether *ORMDL3* knockdown in epithelial cells also influences the inflammatory response to live viral infection, we silenced *ORMDL3* in A549 and BEAS-2B epithelial cells and infected these cells with HRV16, one of the most common rhinoviruses. We then evaluated the effects of knockdown on HRV16-induced IL-6 and IL-8 release as compared to the non-infection control in each group. Basal levels of IL-6 and IL-8 in control A549 cells transfected with scramble oligo were 0.59 ± 0.37 pg/mL and 5.2 ± 0.20 ng/mL, respectively, and HRV16 markedly induced IL-6 (0.59 ± 0.37 pg/mL) and only weakly induced IL-8 (5.2 ± 0.20 ng/mL). In contrast, *ORMDL3* knockdown cells did not enhance IL-6 and IL-8 production after HRV16 inoculation (IL-6 [HRV16/no-inoculation]: 2.2 ± 0.89/2.7 ± 2.3 pg/mL; IL-8 [HRV16/no-inoculation]: 6.2 ± 0.12/6.8 ± 0.46 pg/mL). Basal levels of IL-6 and IL-8 in control BEAS-2B cells transfected with scramble oligo were 2.8 ± 0.11 pg/mL and 16. 4 ± 2.6 pg/mL, respectively, and HRV16 markedly induced IL-6 (246 ± 31.8 pg/mL) and IL-8 (239 ± 49.2 pg/mL). In contrast, in *ORMDL3* knockdown cells, HRV16 induced IL-6 and IL-8 production but the induction levels are relatively lower than those in control cells (IL-6 [HRV16/no-inoculation]: 231 ± 28.8/4.1 ± 1.2 pg/mL; IL-8 [HRV16/no-inoculation]: 156 ± 45.5/ 17.0 ± 3.0 pg/mL). To demonstrate the difference clearer, we calculated the induction ratio [HRV16/non-inoculation] in each cytokine. As shown in Fig. 6, HRV16-dependent induction of IL-6 compared with base line was significantly lower in *ORMDL3* knocked-down A549 cells (*P* < 0.05) and also bronchial epithelial cell line BEAS-2B (*P *< 0.05). For IL-8, levels were also somewhat reduced in the context of *ORMDL3* knockdown, but this did not achieve statistical significance in either A549 cells (*P* = 0.20) or BEAS-2B cells (*P* = 0.13). These data are broadly consistent with our observations from the viral mimic poly I:C and together confirm that ORMDL3 is involved in HRV16-dependent cytokine production as well as poly I:C dependent cytokine release.

## Discussion

Viral respiratory infections show robust association with asthma exacerbations in the community. Human rhinovirus, in particular, is the predominant virus associated with asthma attacks [16], although other viral pathogens such as RSV and influenza may also contribute to the disease [17, 18].

In this report, we generated *ORMDL3* knockdown and *ORMDL3* over-expressing epithelial cells and investigated the inflammatory response after administration of poly I:C. Knockdown of *ORMDL3* and over-expression had little influence of *TLR3* transcript levels in A549 cells. However*,* in both A549 and NHBE cells, knockdown of *ORMDL3* led to a blunted poly I:C-induced inflammatory response. Conversely *ORMDL3* over-expression led to an enhanced poly I:C-induced inflammatory response in these cells. The reduction of cytokine release by *ORMDL3* knockdown was also confirmed in HRV16-infected cells. Thus, the results suggest a key role for *ORMDL3* in the host response to viral infection.

The IL-23/Th17 pathway is a central component of cellular immunity and IL-17A is a signature cytokine of this pathway [19]. IL-17A is widely reported to regulate chronic inflammatory diseases, including respiratory diseases such as asthma [20]. We show here that IL-17A induces an inflammatory response in epithelial cells, and that *ORMDL3* regulates this response. In this report, we did not observe an impact of *ORMDL3* on the inflammatory response to combined stimulations of poly I:C and IL-17A; suggesting that *ORMDL3* participates in multiple pathways in epithelial cells.

Multi-transcript profiling showed that overexpression of *ORMDL3* up-regulates *TRIF*, suggesting regulation of the inflammatory response through the NF-κB pathway. Following poly I:C stimulation control cells showed significant decreases in the transcript levels of *IFNB* and *TRIF*, and increases in the cytokines *IL1*, *IL8* and *IL6*, indicating that poly I:C is a potent inflammatory inducer. Knockdown of *ORMDL3* blunted the *TRIF* response to poly I:C, confirming that *ORMDL3* works in the NF-κB pathway [6]. Our results indicate that *ORMDL3* is an important regulator of the inflammatory response to poly I:C in epithelial cells, and that this impact may not be restricted to the TLR3 signalling pathway—since we did not observe the significant differences in the expression of *IFNB* between *ORMDL3* knockdown and control cells, or between *ORMDL3* over-expressing cells and control cells.

Viral respiratory tract infections are an established risk factor for asthma and can cause symptom relapse or exacerbations. The mechanisms underpinning these features are not known. Viral infection could contribute to asthmatic symptoms and/or change airway physiology [21]. TLRs transduce the infection signal through MAPKs, NF-κB, and IRF3, which induce the transcription of proinflammatory cytokines and type I interferon [22]. TRIF signalling also has both protective and pathologic roles in several chronic inflammatory disease conditions [23].

Toll-like receptors (TLRs) play important roles in the induction of innate antiviral immune responses to viral infections as well as in in development, homeostasis and injury repair [24]. A total of ten TLRs have been identified in humans. Of these, TLR3, first characterized as a regulator of anti-viral responses [25, 26]. TLR3 is assembled in the endoplasmic reticulum, from where it is recruited to endosomes by the transmembrane protein UNC93B1 [27]. TLR3 directly recruits TRIF to its TIR domain to initiate signalling, leading to the activation of the serine/threonine kinase TBK-1, which in turn phosphorylates interferon regulatory factor 3 (IRF3) [24]. TLR3 signalling via TRIF also activates NF-κB [28]. As such, treatment of wounded skin with poly I:C; a dsRNA mimic, significantly reduces recovery time in both humans and mice [29]. In this study, we did not observe that ORMDL3 directly influenced *TLR3* and *IFNB* transcript level after poly I:C stimulation, but it regulated transcript levels of *TRIF* and several cytokine genes. ORMDL3 may work in multiple signaling pathways to regulating viral infection through ER stress, sphingolipid metabolism, glycolysis and other mechanisms [10].

In this report, we found *ORMDL3* knockdown epithelial cells release less IL-6 after HRV16 infection, consistently confirm the results from poly I:C stimulations in *ORMDL3* knockdown cells. In an experiment with HRV14, another HRV strain used ICAM-1 as receptor for entrance of epithelial cells, IL-6 and IL-8 levels were found to increase by the infection [30]. HRV induced the cytokines release may partly activate an NF-kB-dependent transcriptional activation pathway [31].

This is the first report on the role of *ORMDL3* in the poly I:C stimulation response in epithelial A549 and NHBE cells. We recognise several limitations. Firstly, including use of a restricted (24 h) time course following poly I:C stimulation, and a synthetic mimic of viral infection. Secondly, HRV16 is less infectious to A549 and BEAS2B cells in monolayer cell sheet underneath culture media compare with primary epithelial cells, and high level of inoculum, which is not relevant to the clinical condition, was required to induce IL-6 and IL-8. HRV16 does not replicate well in these cells compared with primary epithelial cells, either. To solve these problems, we will use air–liquid interface cultured human bronchial epithelium, which is known to be well infected with HRV16 [32], The viral attachment, replication and inflammatory response of HRV16 infection for *ORMDL3* knockdown epithelial cells need to be future in-depth investigation. Nevertheless, the observations presented here have significant clinical implications, and warrant further investigation focusing on the precise mechanisms through which *ORMDL3* moderates the inflammatory response to viral infection.

## Conclusions

*ORMDL3* significantly influences release of cytokines following poly I:C stimulation in airway epithelial cells. *ORMDL3* regulates the poly I:C-induced inflammatory response via mechanisms independent of the TLR3 pathway.

## Supplementary Information

**Additional file 1.** Western Blotting Images of ORMDL3.

## Data Availability

All data are available from the corresponding author on reasonable request.

## Abbreviations

ORMDL3: Oroscomucoid 3
poly I:C: Polyinosinic-polycytidylic acid
dsRNA: Double-stranded RNA
TLR3: Toll-like receptor 3
NHBE cells: Normal human bronchial epithelial cells
IFNB: Interferon-ß
HRV: Human rhinovirus
ICAM1: Intercellular adhesion molecule 1
RSV: Respiratory syncytial virus
ER: Endoplasmic reticulum
SPT: Palmitoyltransferase
SERCA: Sarcoplasmic/endoplasmic reticulum calcium ATPase
MOI: Multiplicity of infection
NF-κB: Nuclear factor kappa-B
