# Supplementary material for: ORMDL3 regulates poly I:C induced inflammatory responses in airway epithelial cells
Source: BMC Pulm Med. 2021 May 17;21:167. doi: 10.1186/s12890-021-01496-5 (PMC8127224; doi:10.1186/s12890-021-01496-5)
Supplement: Supplementary file 1 — Additional file 1. Western Blotting Images of ORMDL3. [file 12890_2021_1496_MOESM1_ESM.docx]

Supplemental Figure 1

*ORMDL3* Knockdown western Image:

1, ORMDL3 knockdown A549 cells

2, Control A549 cells

3, 4, Other cells

The marker was Amersham™ ECL™ Rainbow™ Marker

The images from the red box were cropped for the manuscript

ORMDL3 (size: 17.5 kDa) β-actin (size: 42 kDa)

1 2 3 4 Marker 1 2 3 4 Marker


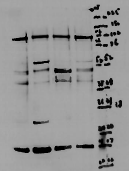

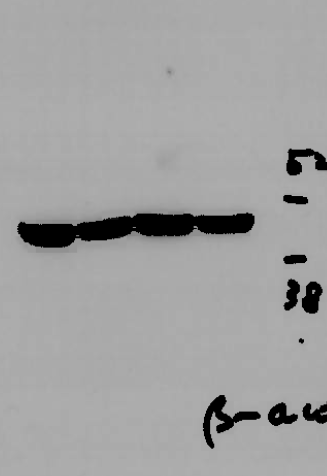

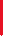

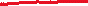

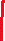

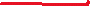

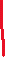

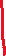

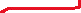

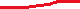


1

*ORMDL3* over-expression western Image:

1, Other cells

2, ORMDL3 over-expression A549 cells

3, Control A549 cells

4, Other cells

The marker was Invitrogen™ Novex™ Sharp Pre-stained Protein Standard

The images from the red box were cropped for the manuscript

ORMDL3 (size: 17.5kDa) β-actin (size: 42kDa)

M 1 2 3 4 M 1 2 3 4


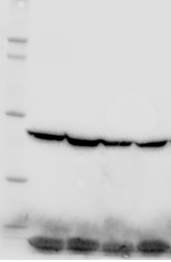

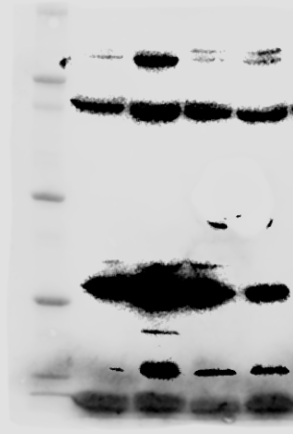

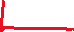

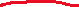

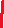

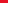

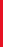

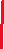

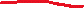

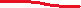


1
